# Supplementary material for: An ethical visualization of the NorthCOVID-19 model
Source: PeerJ Comput Sci. 2022 May 16;8:e980. doi: 10.7717/peerj-cs.980 (PMC9137975; doi:10.7717/peerj-cs.980)
Supplement: Supplemental Information 1 [file peerj-cs-08-980-s001.pdf]

PART 1

1. I think that I would like to use the NorthCOVID-19 system frequently

Strongly Disagree O O O O O Strongly Agree

1 2 3 4 5

2. I found the NorthCOVID-19 system unnecessarily complex

Strongly Disagree O O O O O Strongly Agree

1 2 3 4 5

3. I thought the NorthCOVID-19 system was easy to use

Strongly Disagree O O O O O Strongly Agree

1 2 3 4 5

4. I think that I would need the support of a technical person to be able to use the NorthCOVID-19 system

Strongly Disagree O O O O O Strongly Agree

1 2 3 4 5

5. I found the various functions in the NorthCOVID-19 system were well integrated

Strongly Disagree O O O O O Strongly Agree

1 2 3 4 5

6. I thought there was too much inconsistency in the NorthCOVID-19 system

Strongly Disagree O O O O O Strongly Agree

1 2 3 4 5

7. I would imagine that most people would learn to use the NorthCOVID-19 system very quickly

Strongly Disagree O O O O O Strongly Agree

1 2 3 4 5

8. I found the NorthCOVID-19 system very cumbersome to use

Strongly Disagree O O O O O Strongly Agree

1 2 3 4 5

9. I felt very confident using the NorthCOVID-19 system

Strongly Disagree O O O O O Strongly Agree

1 2 3 4 5

10. I needed to learn a lot of things before I could get going with the NorthCOVID-19 system

Strongly Disagree O O O O O Strongly Agree

1 2 3 4 5

PART 2

1. I feel confident in my understanding of the video output

Strongly Disagree O O O O O Strongly Agree

1 2 3 4 5

2. In comparison to the video output, I found that the NorthCOVID-19 website output is easier to understand

Strongly Disagree O O O O O Strongly Agree

1 2 3 4 5

3. In the video output, I found the audio that accompanied it to be helpful in understanding the results

Strongly Disagree O O O O O Strongly Agree

1 2 3 4 5

4. I feel the visuals/graphs provided in the video improved the comprehension of the data

Strongly Disagree O O O O O Strongly Agree

1 2 3 4 5

5. I feel the information provided in the video is enough for me to make an informed decision

Strongly Disagree O O O O O Strongly Agree

1 2 3 4 5
